# Supplementary material for: Decoding spatiotemporal transcriptional dynamics and epithelial fibroblast crosstalk during gastroesophageal junction development through single cell analysis
Source: Nat Commun. 2024 Apr 9;15:3064. doi: 10.1038/s41467-024-47173-z (PMC11004180; doi:10.1038/s41467-024-47173-z)
Supplement: Supplementary file 1 — Supplementary Information [file 41467_2024_47173_MOESM1_ESM.pdf]

Supplementary Information

Decoding Spatiotemporal Transcriptional Dynamics and Epithelial Fibroblast Crosstalk during Gastroesophageal Junction Development through Single Cell Analysis

Naveen Kumar[1,2,8], Pon Ganish Prakash[2,8], Christian Wentland[2], Shilpa Mary Kurian[2], Gaurav Jethva[2], Volker Brinkmann[3], Hans-Joachim Mollenkopf[3], Tobias Krammer[4], Christophe Toussaint[4], Antoine-Emmanuel Saliba[4,5], Matthias Biebl[6], Christian Jürgensen[7], Bertram Wiedenmann[7], Thomas F Meyer[3], Rajendra Kumar Gurumurthy[2,3,7], Cindrilla Chumduri[1,2,3,7,#]

[1] Laboratory of Infections, Carcinogenesis and Regeneration, Medical Biotechnology Section, Department of Biological and Chemical Engineering, Aarhus University, Denmark
[2] Department of Microbiology, University of Würzburg, Würzburg, Germany
[3] Department of Molecular Biology, Max Planck Institute for Infection Biology, Berlin, Germany
[4] Helmoltz Institute for RNA-based Infection Research (HIRI), Helmholtz-Center for Infection Research (HZI), Würzburg, Germany
[5] University of Würzburg, Faculty of Medicine, Institute of Molecular Infection Biology (IMIB), Würzburg, Germany.
[6] Surgical Clinic Campus Charité Mitte, Charité University Medicine, Berlin, Germany.
[7] Department of Hepatology and Gastroenterology, Charité University Medicine, Berlin, Germany.
[8] These authors contributed equally
[#]Corresponding author

Corresponding address:
Prof. Cindrilla Chumduri
Laboratory of Infections, Carcinogenesis and Regeneration,
Medical Biotechnology section,
Department of Biological and Chemical Engineering,
Aarhus University,
Gustav Wieds Vej 10C, Buil. 3133
8000 Aarhus C, Denmark
Mail: cindrilla.chumduri@bce.au.dk
cindrilla.chumduri@uni-wuerzburg.de

This file includes:
Supplementary Figures 1 to 9
Description of Supplementary Data 1 to 10

Supplementary Fig.1

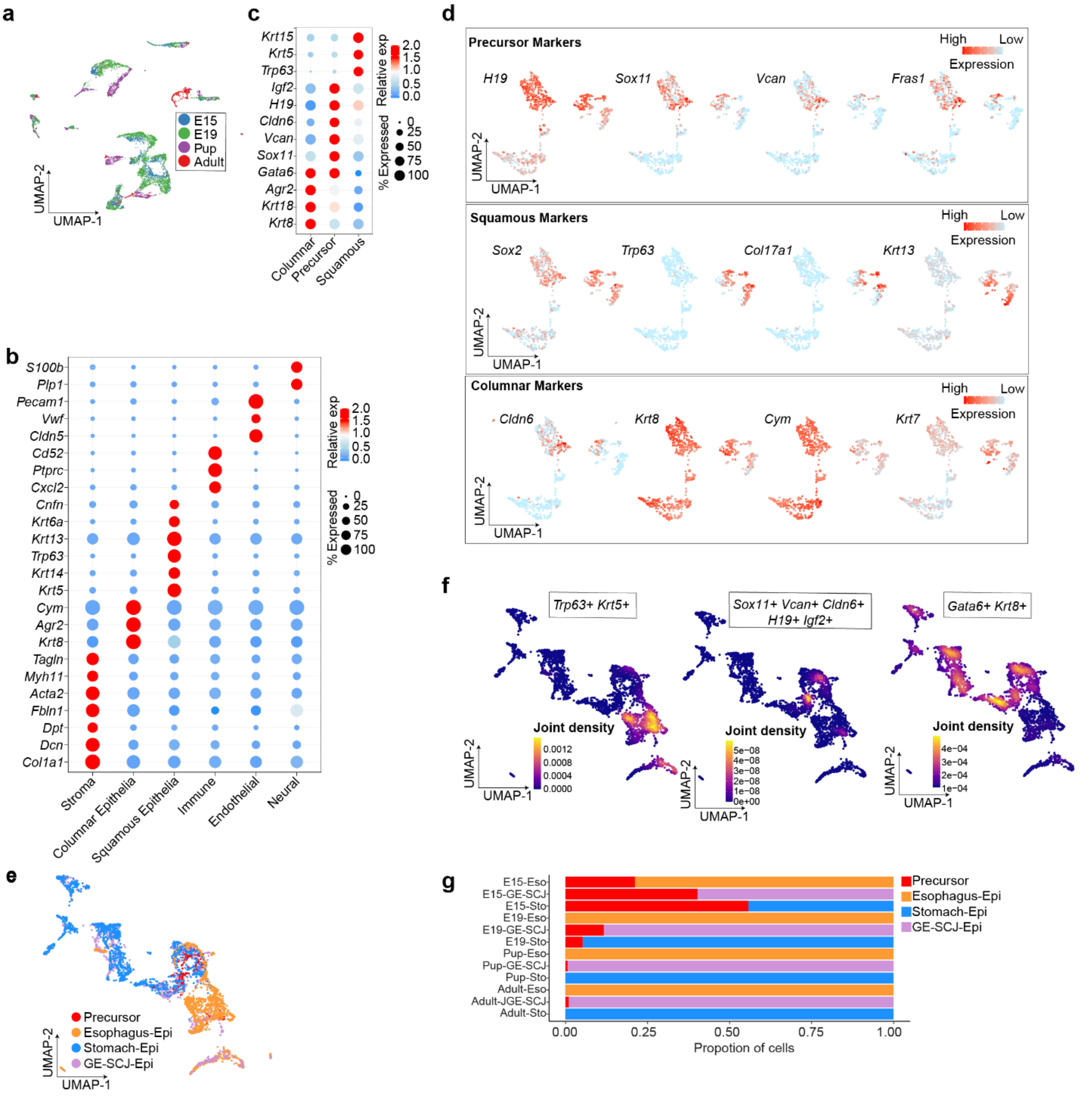

Supplementary Fig. 1. Single-cell epithelial landscape of GE-SCJ. a UMAP depicting the distribution of identified cell clusters over time; each dot represents a single cell, colored by time point, including E15, E19, pup, and adult. **b** Dot plot showing the expression of canonical markers associated with each identified cell type. **c** Dot plot depicting the expression of gene markers used in identifying GE-SCJ epithelial cell types; dot size represents the percentage of cells expressing a particular gene; color bar indicates the intensity of scaled mean expression levels from high (red) to low (blue) (**b-c**). **d** Feature plot showing the expression of indicated genes corresponding to GE-SCJ epithelial cell types. **e** UMAP showing epithelial cell clusters from esophagus, stomach and GE-SCJ samples, cells colored by tissue; highlighted precursor

cells from GE-SCJ are shown in red. **f** Joint gene-weighted density estimation of markers corresponding to squamous, columnar and precursor epithelial cells. **g** Bar plot illustrating the distribution of precursor epithelial cells across tissue and time points.

Supplementary Fig. 2

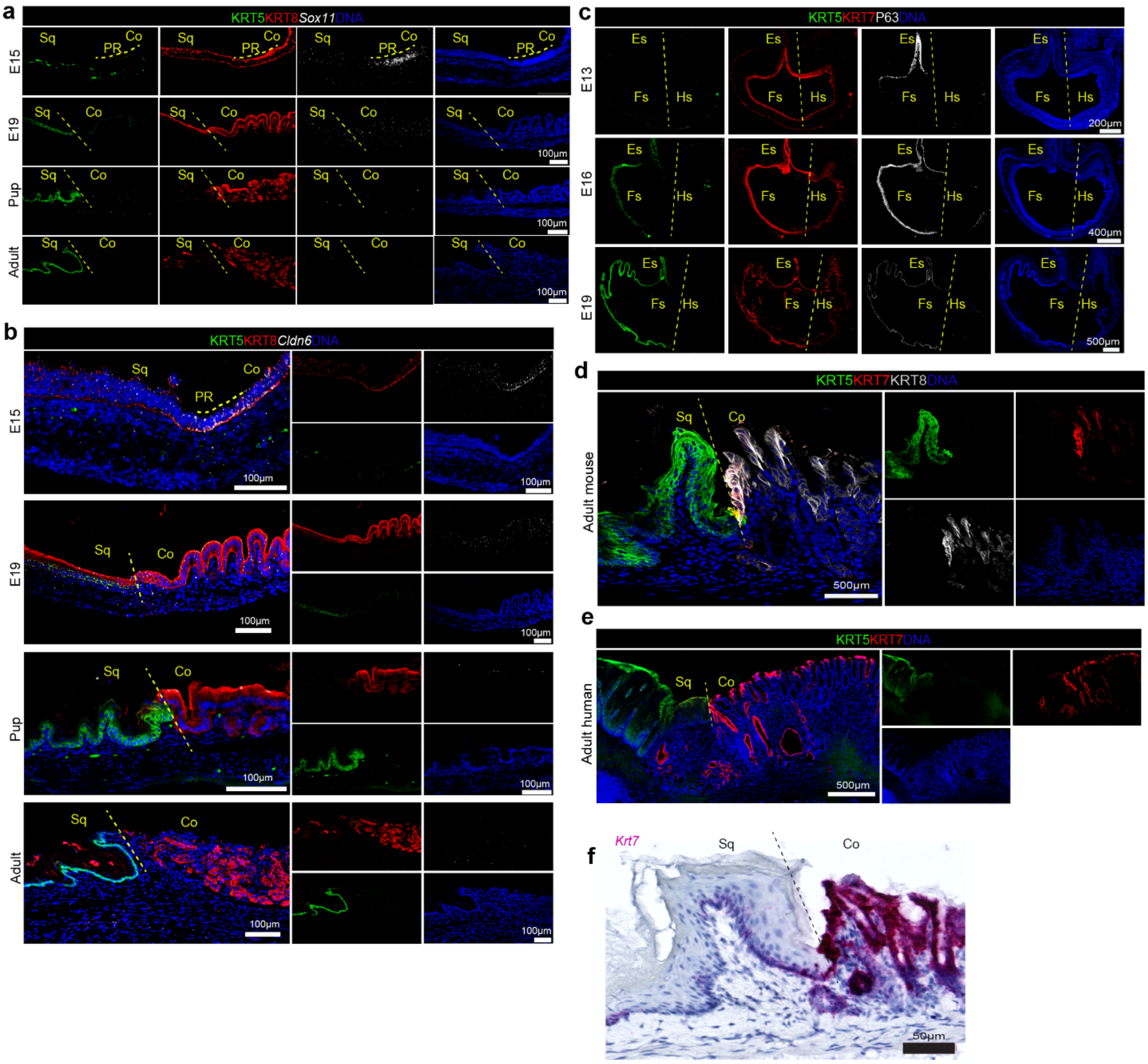

Supplementary Fig. 2. **Characterization of epithelial cells across embryonic to adult GE-SCJ**. **a-b** Individual channels of tiled images of mouse GE-SCJ probed by smRNA-ISH for *Sox11* (white) (**a**), *Cldn6* (white) (**b**) and co-immunostained for KRT5 (green), KRT8 (red). **c** Individual channels of tiled images of the entire stomach, including distal esophagus, immunostained with KRT5 (green), KRT7 (Red), P63 (white). **d-e** Tiled images of an adult mouse (**d**) and human (**e**) GE-SCJ tissue immunostained for indicated marker proteins. **f** smRNA-ISH images of mouse GE-SCJ tissue probed for *Krt7*, nuclei (blue). Sq, Co, PR, Es, Fs, Hs indicate squamous epithelia, columnar epithelia, precursor cell region, esophagus, forestomach, and hind stomach respectively. All images are representative of three biological replicates.

Supplementary Fig. 3

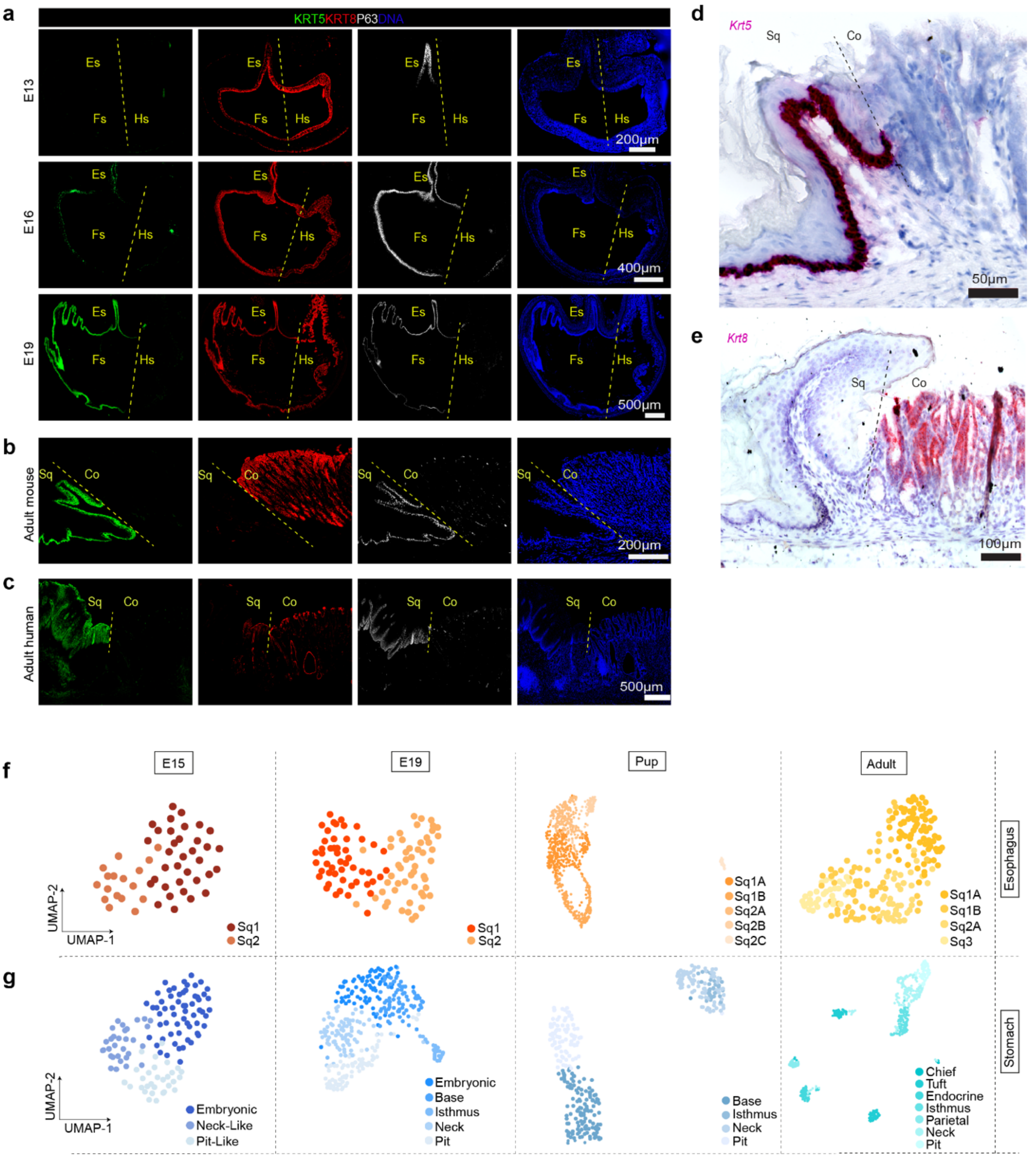

Supplementary Fig. 3. Development of epithelial subpopulation across embryonic to adult gastroesophageal epithelia. **a-c** Individual channels of tiled images of tissue sections from the entire stomach, including distal esophagus E13, E16, and E19 mice (**a**); GE-SCJ region of the adult mouse (**b**) and human (**c**) immunostained for KRT5 (green), KRT8 (Red), P63 (white), and nuclei (blue). **d-e** smRNA-ISH images of mouse GE-SCJ tissue probed for *Krt5* (**d**) and *Krt8* (**e**). Nuclei are labeled in blue. Sq, Co, Es, Fs, and Hs indicate squamous epithelia, columnar epithelia, esophagus, forestomach, and hind stomach, respectively. **f-g** UMAP projection of

epithelial cells from the esophagus (**f**) and stomach (**g**) samples at four different time points; each dot represents a single cell and is colored by epithelial subtypes. Images are representative of three biological replicates in (**a-e**).

Supplementary Fig. 4

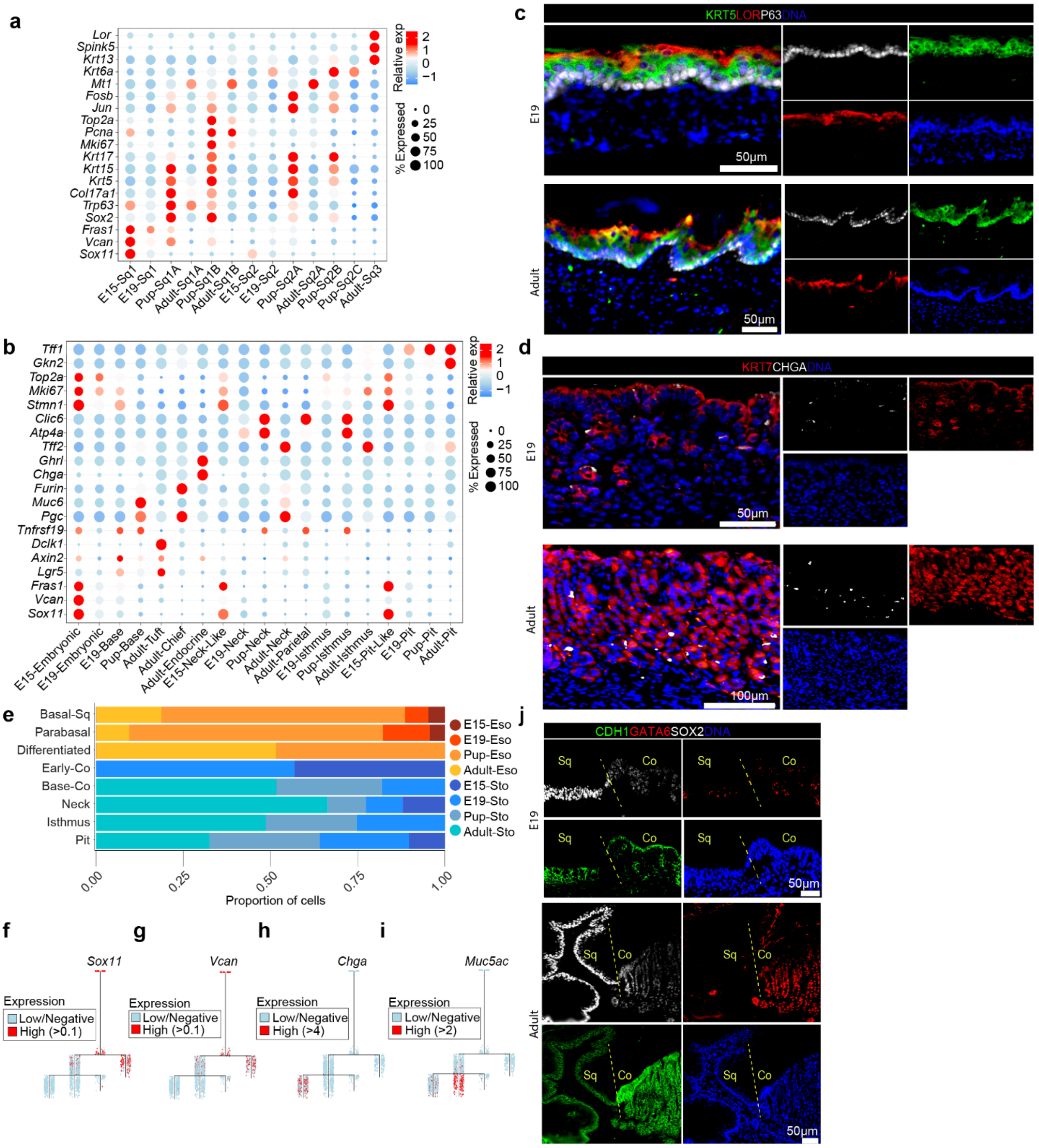

Supplementary Fig. 4. Epithelial subpopulation across embryonic to adult gastroesophageal tissue. a-b Dot plot depicting the expression of canonical markers for esophageal squamous epithelial (**a**) and stomach columnar epithelial (**b**) cell types ordered from embryonic to adult time points. The dot size represents the percentage of cells expressing a particular gene; the color bar indicates the intensity of scaled mean expression levels from high (red) to low (blue). **c** Confocal images of the mouse esophagus at E19 and adult immunostained with KRT5 (green), LOR (red), P63 (white) and nuclei (blue). **d** Confocal images of the mouse stomach at E19 and adult immunostained for KRT7 (red), CHGA (white) and nuclei (blue). **e** Bar plot

showing the relative proportion of squamous and columnar epithelial cell subtypes at different time points. **f-i** URD differentiation tree of stomach epithelial cells highlighting the expression of early embryonic markers *Sox11* (**f**), *Vcan* (**g**) and late differentiation markers *Chga* (**h**), *Muc5ac* (**i**); each dot represents a single cell, with color indicating expression levels as high (red) and low/negative (blue). **j** Individual channels of confocal images of the E19 and adult mouse GE-SCJ immunostained for CDH1 (green), GATA6 (red), SOX2 (white) and nuclei (blue). Sq and Co, indicate squamous and columnar epithelia, respectively. Images are representative of three biological replicates in (**c-d, j**).

Supplementary Fig. 5

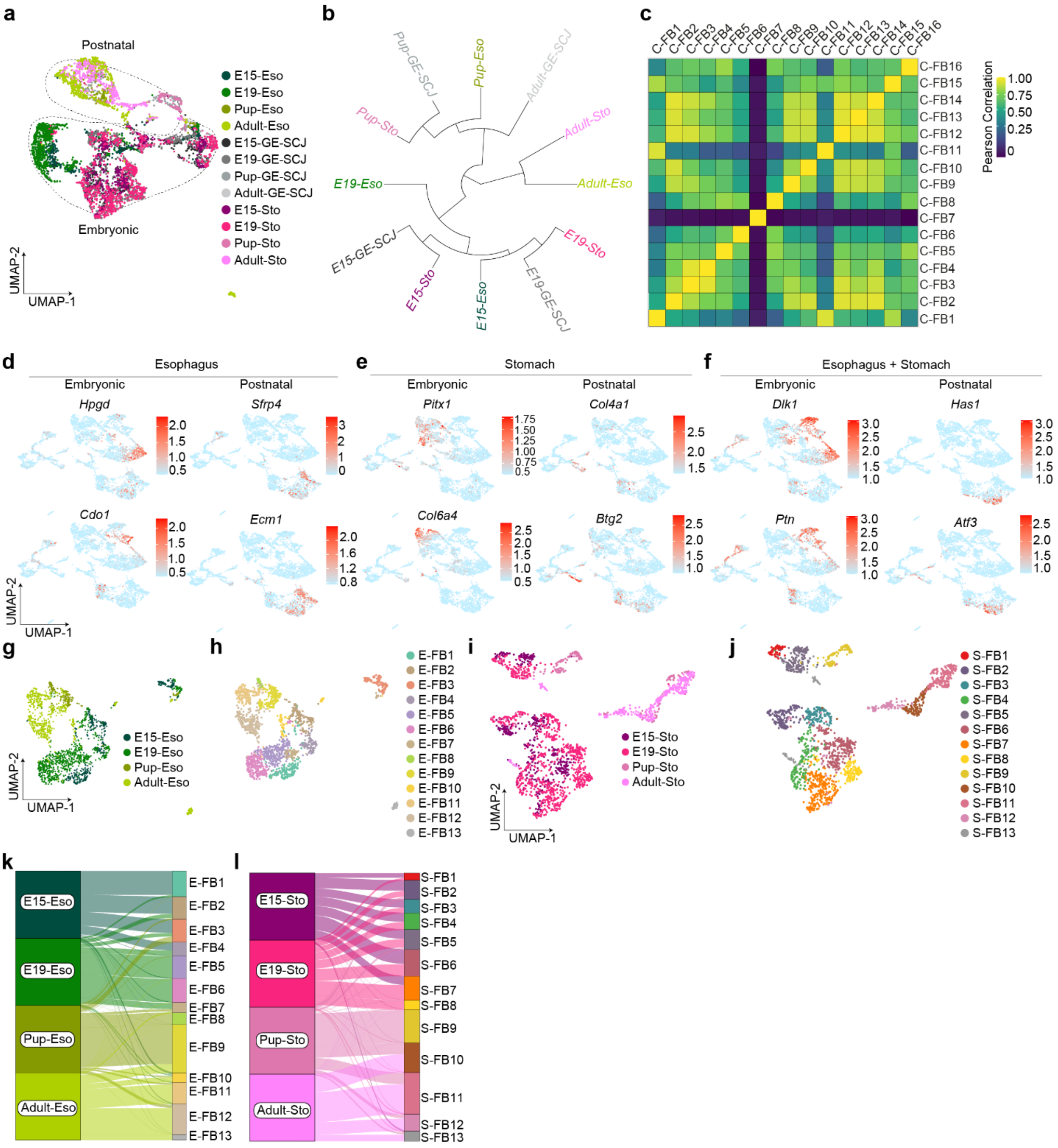

Supplementary Fig. 5. Evolution of fibroblast microenvironment underlying gastroesophageal tissue from embryonic to adult stages. a UMAP of fibroblast cell clusters from all tissue regions (including GE-SCJ) at different developmental stages, colored by tissue type and time point. **b** Circular dendrogram showing the similarity between clusters as in (**a**); font color denotes tissue type and time point. **c** Heatmap visualization of Pearson correlation matrix between the identified fibroblast subclusters. **d-f** Feature plot showing normalized gene expression levels in both exclusive (**d, e**) and shared (**f**) fibroblast population between esophagus and stomach. **g-h** UMAP showing fibroblast cell clusters of only esophagus; colored

by time point (**g**) and cluster annotation (**h**). **i-j** UMAP showing stomach fibroblast cell clusters; colored by time point (**i**) and cluster annotation (**j**). **k-l** Sankey plots showing the contribution of fibroblast cells from the esophagus (**k**) and stomach (**l**) samples at each time point to their respective subclusters, as shown in (**h, j**).

Supplementary Fig. 6

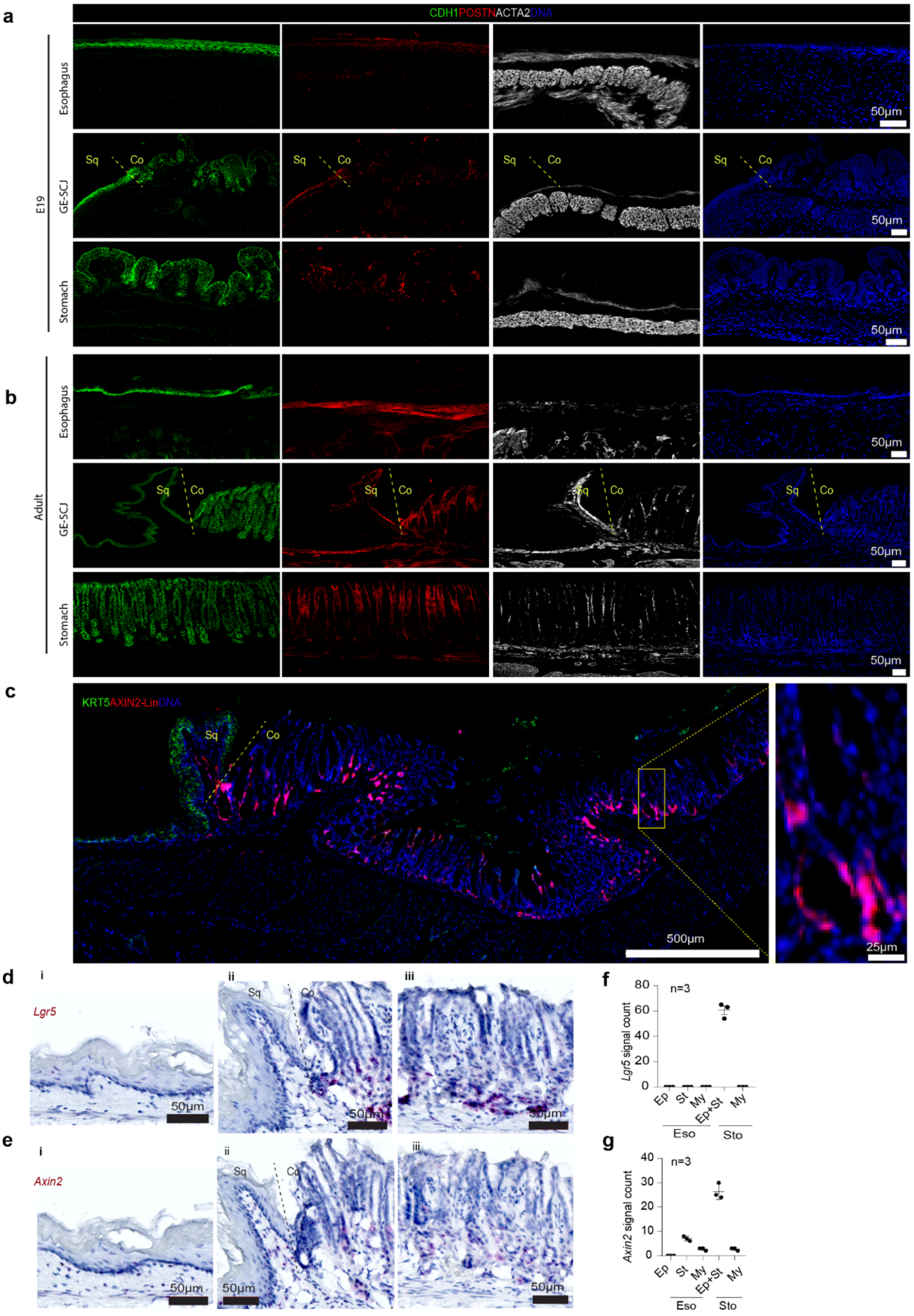

Supplementary Fig. 6. WNT microenvironment in GE-SCJ. a-b Individual channels of tiled images of esophagus, GE-SCJ and stomach tissue sections from E19 (**a**) and adult (**b**) immunostained with CDH1 (green), POSTN (red), ACTA2 (white) and nuclei (blue). Images are representative of n=3 mice. **c** Tiled images of GE-SCJ sections from *Axin2-CreERT2/Rosa26-tdTomato* mice co-immunostained for KRT5 (green), AXIN2 lineage traced cells marked by Tdtomato (red), and nuclei (blue). Inset images depict enlarged portion Axin2 lineage traced stomach gland epithelial cell. **d-e** smRNA-ISH images for *Lgr5* (**d**) and *Axin2* (**e**) in the mouse esophagus tissue (i), at GE-SCJ (ii), and in stomach glands (iii), nuclei (blue). **f-g** Quantification of *Lgr5* (**f**) and *Axin2* (**g**) signal counts in epithelia (Ep), stroma (St), and myofibroblast (My) in the esophageal and stomach tissue regions. n = number of signal counts derived from three non-overlapping 100 μm^2 regions of esophagus and stomach tissues (**f-g**). Data are presented as mean values +/- SEM. Sq and Co, indicate squamous and columnar epithelia, respectively. Images are representative of three biological replicates in (**a-e**). For (**f-g**), source data are provided as a Source Data file.

Supplementary Fig. 7

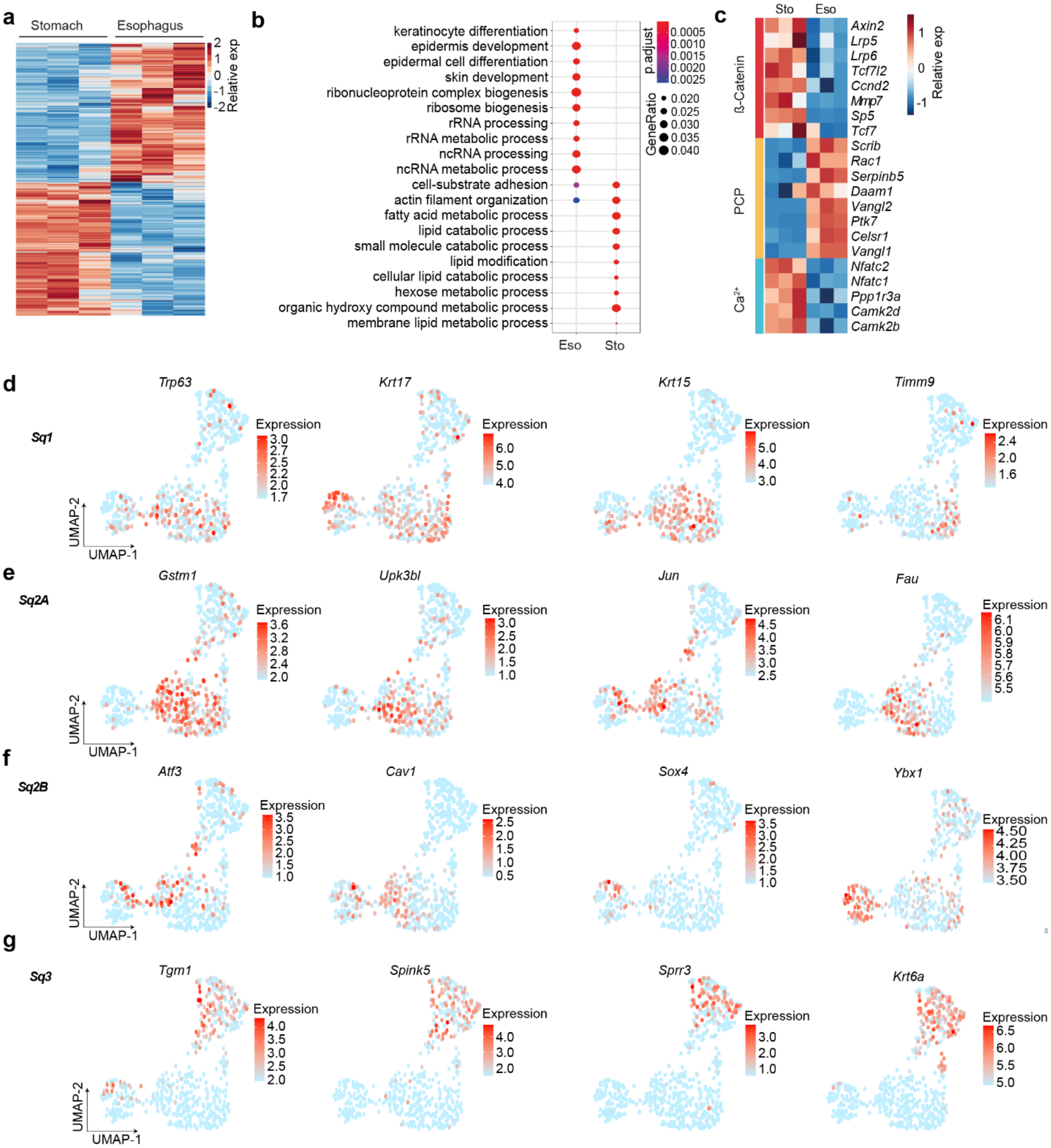

Supplementary Fig. 7. Organoid transcriptomics revealing functional differences between esophagus and stomach. a Heatmap showing DEG in esophagus versus stomach organoids. Columns represent organoids derived from individual mice. The color bar represents z-scored gene expression. **b** Top 10 enriched GO terms associated with DEG between esophageal and stomach organoids. **c** Heatmap showing expression of differentially regulated Wnt signaling pathway genes in esophagus versus stomach organoids. Columns represent organoids derived from individual mice. The color bar represents z-scored gene expression. **d-g**

Normalized expression values of selected markers on UMAP representing esophageal epithelial subclusters as in Fig 5s, for Sq1 (**d**), Sq2A (**e**), Sq2B (**f**), Sq3A, and Sq3B (**g**).

Supplementary Fig. 8

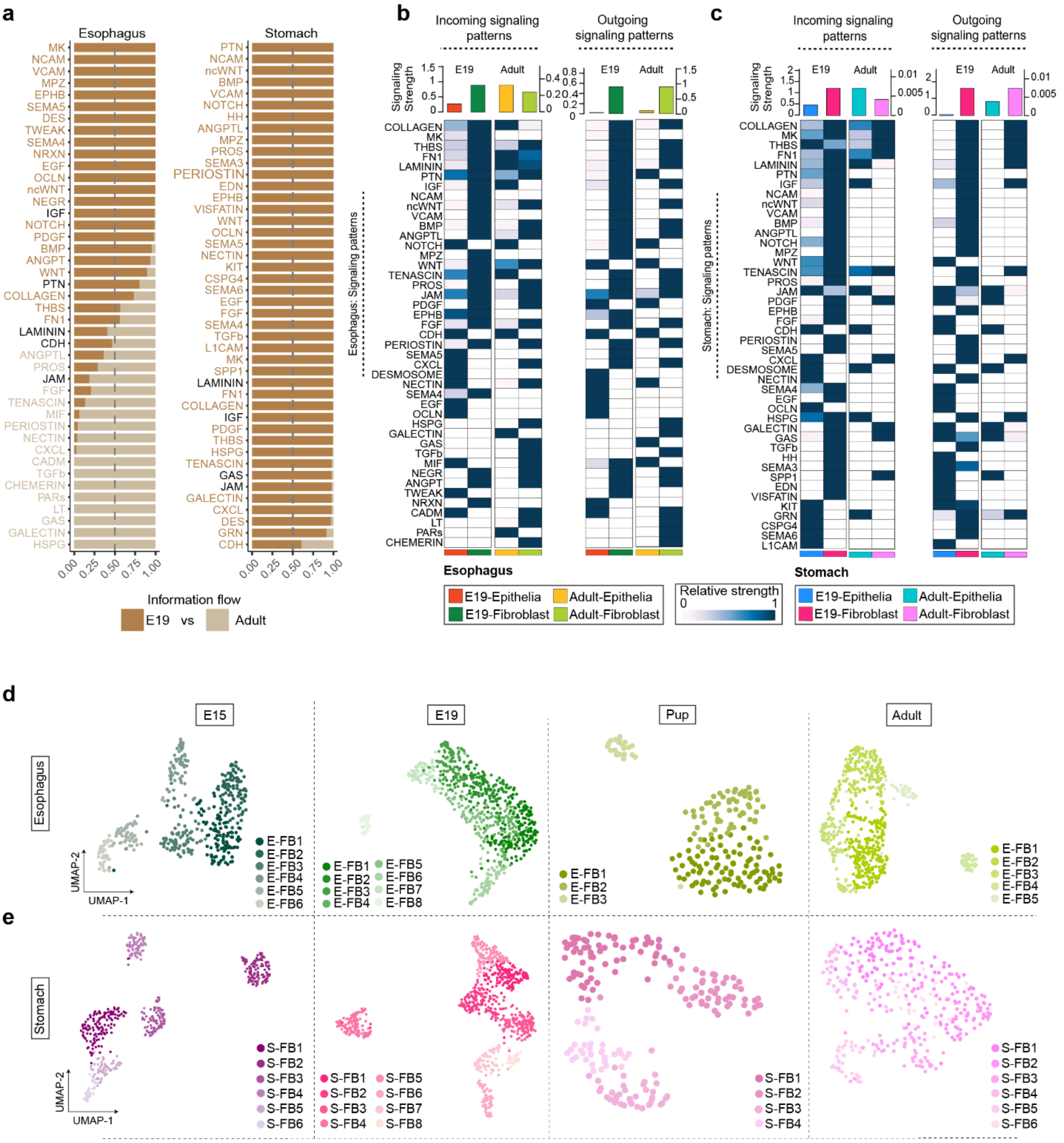

Supplementary Fig. 8. Intercellular signaling patterns between epithelial and fibroblast cells in pre- and postnatal gastroesophageal tissue. a Stacked bar graph showing significant signaling pathways ranked based on their differences of overall interaction strength within inferred networks between E19 and adult time points in esophagus and stomach; signaling pathways colored in dark brown are enriched in E19 stage, and those colored light brown are enriched in the adult time point. **b-c** Heatmap highlighting the changes in incoming and outgoing signaling patterns associated with fibroblast and epithelial compartments of the esophagus (**b**) and stomach (**c**). Scale bar denotes the relative signaling strength (row-scaled

values) of a pathway across cell types. The relative strength of a pathway is calculated by normalizing each row of values to fall within the range 0-1 and depicted as low (white) to high (dark blue). Colored bar plot on top depicts the total signaling strength of each cell type by summarizing all pathways in the heatmap. **d-e** UMAP showing fibroblast cell clusters of the esophagus (**d**) and stomach (**e**) at individual time points. Each dot represents a single cell and is colored by cluster annotation.

Supplementary Fig. 9

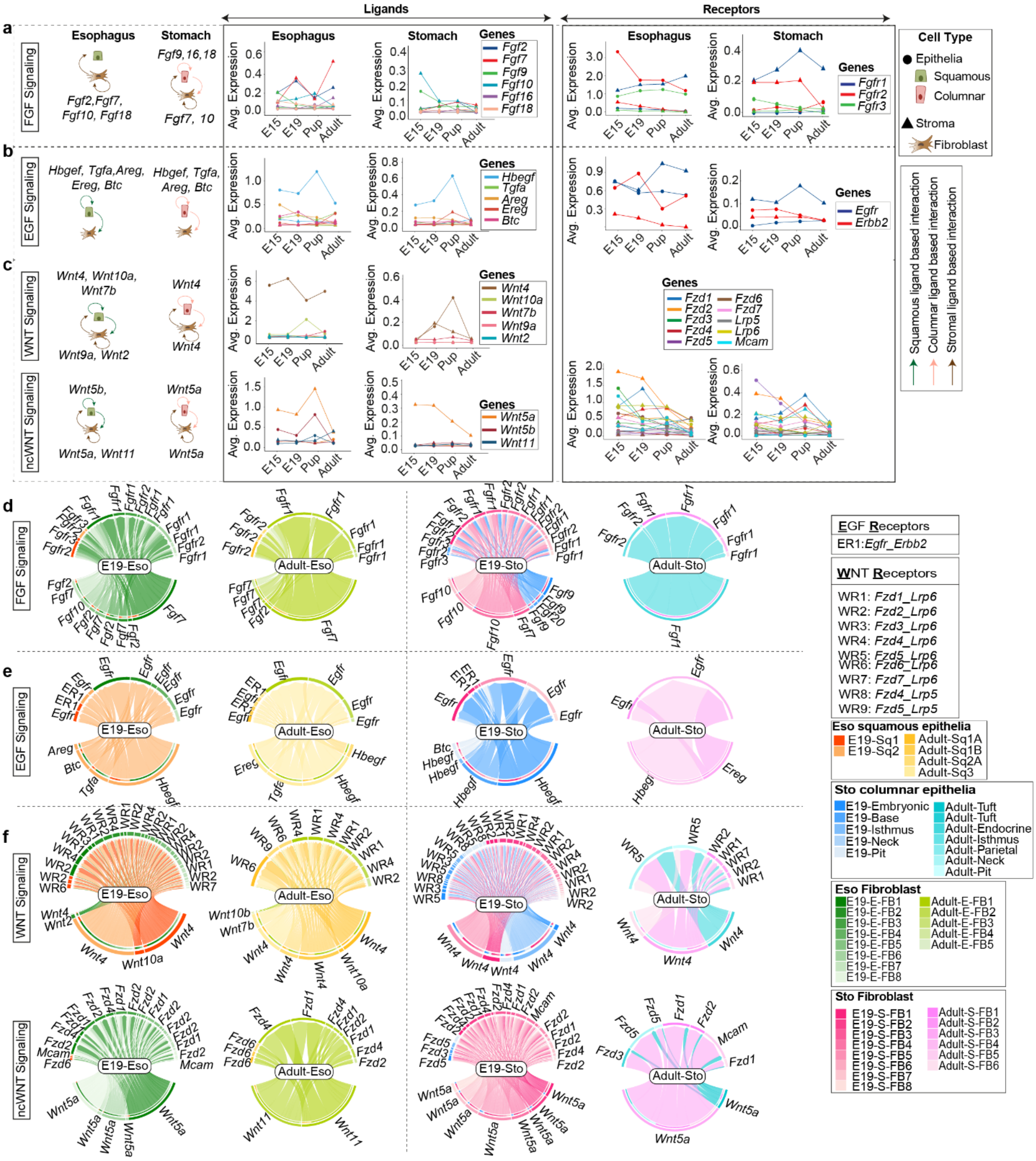

Supplementary Fig. 9. Cell-cell interaction dynamics in pre- and postnatal gastroesophageal tissue: a-c Graphical abstract of tissue-specific signaling directions between epithelial and fibroblast (left); trend plot showing the mean expression dynamics of key ligands and receptors over time (right); for the following signaling pathways of interest: FGF (a), EGF (b), WNT and ncWNT (**c**). **d-f** Chord diagrams depicting inferred cell-cell communications mediated by multiple significant ligand-receptors between epithelia and fibroblast in esophagus and stomach at E19 and adult time points for FGF (**d**), EGF (**e**), WNT and ncWNT

(**f**) pathways; In lower half of the circos plot, outer bars colored by signal sending cell groups; inner bars colored by proportion of receiving cell groups; edges colored by signaling senders.

Supplementary Data 1

List of antibodies used in this study.

Supplementary Data 2

Related to Figure 1f. Scaled expression values for differentially expressed genes among GE-SCJ epithelial subclusters, with cells ordered based on diffusion map (DM) from left to right as shown in Figure 1e.

Supplementary Data 3

Related to Figure 2k. Differentially regulated genes across epithelial stem cell compartments of esophagus and stomach samples from the embryonic to adult time points. Statistical significance was determined using the Wilcoxon rank-sum test on genes detected in 15% of barcodes, showing a minimum 0.25-fold difference (log scale) across clusters via the Seurat function.

Supplementary Data 4

Related to Figure 2l. Scaled activity scores of variable transcription factors (TF) across epithelial stem cell compartments of esophagus and stomach samples from the embryonic to adult time points.

Supplementary Data 5

Related to Figure 3f. Differentially expressed genes across combined fibroblast subclusters. Statistical significance was determined using the Wilcoxon rank-sum test on genes detected in 15% of barcodes, showing a minimum 0.25-fold difference (log scale) across clusters via the Seurat function.

Supplementary Data 6

Related to Figure 4c: Data distribution and error bar metrics, which include standard deviation (SD), standard error of mean (SEM), confidence interval (CI) 95%, and quartile (Q) values for genes *Rspo3* and *Dkk2*.

Supplementary Data 7

Related to Supplementary Figure S7a. Scaled averaged gene expression values of unique genes (Calculated by averaging the expression values of genes corresponding to two or more probes).

Supplementary Data 8

Related to Supplementary Figure S7b. Results from the GO Biological Process Over-enrichment analysis for genes differentially expressed between esophagus and stomach. Statistical significance was calculated using hypergeometric test and adjusted for multiple testing (Benjamini–Hochberg method) using ClusterProfiler.

Supplementary Data 9

Related to Figure 6a. Z-scored gene set enrichment scores of both fibroblasts and epithelial cells of the esophagus and stomach for pathways of interest from embryonic to adult time points.

Supplementary Data 10

Related to Figure 7a-c and Supplementary Figure 9a-c. Data distribution and error bar metrics, which include standard deviation (SD), standard error of mean (SEM), confidence interval (CI) 95%, and quartile (Q) values for all ligands and receptors.
