# Supplementary material for: Decoding spatiotemporal transcriptional dynamics and epithelial fibroblast crosstalk during gastroesophageal junction development through single cell analysis
Source: Nat Commun. 2024 Apr 9;15:3064. doi: 10.1038/s41467-024-47173-z (PMC11004180; doi:10.1038/s41467-024-47173-z)
Supplement: Supplementary file 3 — Description of Additional Supplementary Files [file 41467_2024_47173_MOESM3_ESM.pdf]

Description of Additional Supplementary Files

File Name: Supplementary Data 1
Description: List of antibodies used in this study.

File Name: Supplementary Data 2
Description: Related to Figure 1f. Scaled expression values for differentially expressed genes among GESCJ epithelial subclusters, with cells ordered based on diffusion map (DM) from left to right as shown in Figure 1e.

File Name: Supplementary Data 3
Description: Related to Figure 2k. Differentially regulated genes across epithelial stem cell compartments of esophagus and stomach samples from the embryonic to adult time points. Statistical significance was determined using the Wilcoxon rank-sum test on genes detected in 15% of barcodes, showing a minimum 0.25-fold difference (log scale) across clusters via the Seurat function.

File Name: Supplementary Data 4
Description: Related to Figure 2l. Scaled activity scores of variable transcription factors (TF) across epithelial stem cell compartments of esophagus and stomach samples from the embryonic to adult time points.

File Name: Supplementary Data 5
Description: Related to Figure 3f. Differentially expressed genes across combined fibroblast subclusters. Statistical significance was determined using the Wilcoxon rank-sum test on genes detected in 15% of barcodes, showing a minimum 0.25-fold difference (log scale) across clusters via the Seurat function.

File Name: Supplementary Data 6
Description: Related to Figure 4c: Data distribution and error bar metrics, which include standard deviation (SD), standard error of mean (SEM), confidence interval (CI) 95%, and quartile (Q) values for genes Rspo3 and Dkk2.

File Name: Supplementary Data 7
Description: Related to Supplementary Figure S7a. Scaled averaged gene expression values of unique genes (Calculated by averaging the expression values of genes corresponding to two or more probes).

File Name: Supplementary Data 8

Description: Related to Supplementary Figure S7b. Results from the GO Biological Process Overenrichment analysis for genes differentially expressed between esophagus and stomach. Statistical significance was calculated using hypergeometric test and adjusted for multiple testing (Benjamini–Hochberg method) using ClusterProfiler.

File Name: Supplementary Data 9
Description: Related to Figure 6a. Z-scored gene set enrichment scores of both fibroblasts and epithelial cells of the esophagus and stomach for pathways of interest from embryonic to adult time points.

File Name: Supplementary Data 10
Description: Related to Figure 7a-c and Supplementary Figure 9a-c. Data distribution and error bar metrics, which include standard deviation (SD), standard error of mean (SEM), confidence interval (CI) 95%, and quartile (Q) values for all ligands and receptors.
