# Supplementary material for: Decoding spatiotemporal transcriptional dynamics and epithelial fibroblast crosstalk during gastroesophageal junction development through single cell analysis
Source: Nat Commun. 2024 Apr 9;15:3064. doi: 10.1038/s41467-024-47173-z (PMC11004180; doi:10.1038/s41467-024-47173-z)
Supplement: Supplementary file 14 — Reporting Summary [file 41467_2024_47173_MOESM14_ESM.pdf]

nature portfolio

Corresponding author(s): Prof. Dr. Cindrilla Chumduri

Last updated by author(s): Mar 12, 2024

Reporting Summary

Nature Portfolio wishes to improve the reproducibility of the work that we publish. This form provides structure for consistency and transparency in reporting. For further information on Nature Portfolio policies, see our Editorial Policies and the Editorial Policy Checklist.

Statistics

For all statistical analyses, confirm that the following items are present in the figure legend, table legend, main text, or Methods section.

n/a	Confirmed	
☐	☒	The exact sample size (*n*) for each experimental group/condition, given as a discrete number and unit of measurement
☐	☒	A statement on whether measurements were taken from distinct samples or whether the same sample was measured repeatedly
☐	☒	The statistical test(s) used AND whether they are one- or two-sided<br>*Only common tests should be described solely by name; describe more complex techniques in the Methods section.*
☒	☐	A description of all covariates tested
☐	☒	A description of any assumptions or corrections, such as tests of normality and adjustment for multiple comparisons
☐	☒	A full description of the statistical parameters including central tendency (e.g. means) or other basic estimates (e.g. regression coefficient) AND variation (e.g. standard deviation) or associated estimates of uncertainty (e.g. confidence intervals)
☐	☒	For null hypothesis testing, the test statistic (e.g. *F*, *t*, *r*) with confidence intervals, effect sizes, degrees of freedom and *P* value noted<br>*Give P values as exact values whenever suitable.*
☒	☐	For Bayesian analysis, information on the choice of priors and Markov chain Monte Carlo settings
☒	☐	For hierarchical and complex designs, identification of the appropriate level for tests and full reporting of outcomes
☒	☐	Estimates of effect sizes (e.g. Cohen's *d*, Pearson's *r*), indicating how they were calculated

Our web collection on statistics for biologists contains articles on many of the points above.

Software and code

Policy information about availability of computer code

Data collection	Microarray image data were analyzed and extracted with the Image Analysis/Feature Extraction software G2567AA v. A.11.5.1.1 (Agilent Technologies).<br>Next-generation sequencing data was collected by Illumina NovaSeq 6000 (10X Chromium libraries)
Data analysis	Graphpad Prism version 8 was used for statistical analysis.<br>R-4.1.1 was obtained from https://cran.r-project.org/<br>Adobe illustrator version 27.4.1<br>ImageJ 1.53c<br>Zen 3.2 (Blue edition) image analysis software<br>Cell Ranger version 6.1.1 software suit (10X Genomics) and Seurat version 4.1.1 were used to process and analyze single cell sequencing data.

For manuscripts utilizing custom algorithms or software that are central to the research but not yet described in published literature, software must be made available to editors and reviewers. We strongly encourage code deposition in a community repository (e.g. GitHub). See the Nature Portfolio guidelines for submitting code & software for further information.

Data

Policy information about availability of data

All manuscripts must include a data availability statement. This statement should provide the following information, where applicable:

- Accession codes, unique identifiers, or web links for publicly available datasets
- A description of any restrictions on data availability
- For clinical datasets or third party data, please ensure that the statement adheres to our policy

Microarray and scRNA-seq data that support the findings of this study have been deposited in the Gene Expression Omnibus (GEO) under accession codes GSE181409, GSE181411 and GSE227412. Raw data associated with figures can be found in the Supplementary Tables respectively.
Previously published scRNA-seq datasets that were re-analysed here are available under accession codes GSE116514, GSE157694, E-MTAB-8662 and CRA002118.
Quantitative data supporting the findings of this study are available within the paper and its supplementary information. Source data underlying the graphical representations in Figures 4e, 4f, 4h, 4i, 5e, 5f, 5i, 5j, Supplementary. Fig. 6f, 6g are provided in the Source Data file.

Research involving human participants, their data, or biological material

Policy information about studies with human participants or human data. See also policy information about sex, gender (identity/presentation), and sexual orientation and race, ethnicity and racism.

Reporting on sex and gender	Male and female patients
Reporting on race, ethnicity, or other socially relevant groupings	Socially constructed variables were not considered in this study.
Population characteristics	Tissue was donated by male and female patients aged 55 to 75
Recruitment	Upon receiving informed consent the biopsies were collected and samples from different donors were randomly assigned to experiments.
Ethics oversight	The Department of Hepatology and Gastroenterology, Charité University Medicine, Berlin, Germany, provided human esophagus, stomach, and Z-line (GEJ) samples. Usage for scientific research was approved by their ethics committee (EA4/034/14); informed consent was obtained from all subjects.

Note that full information on the approval of the study protocol must also be provided in the manuscript.

Field-specific reporting

Please select the one below that is the best fit for your research. If you are not sure, read the appropriate sections before making your selection.

[x] Life sciences [ ] Behavioural & social sciences [ ] Ecological, evolutionary & environmental sciences

For a reference copy of the document with all sections, see nature.com/documents/nr-reporting-summary-flat.pdf

Life sciences study design

All studies must disclose on these points even when the disclosure is negative.

Sample size	Sample size was not predetermined by statistics.
Data exclusions	No data were excluded from the experiments.
Replication	All attempts of replication were successful. All graphs and images represent findings from at least two independent replicates, see figure legends. All micro array analysis represent data from three independent experiments.
Randomization	No specific procedures were carried out for randomization.
Blinding	The investigator was blinded for data collection and quantitative analysis.

Reporting for specific materials, systems and methods

We require information from authors about some types of materials, experimental systems and methods used in many studies. Here, indicate whether each material, system or method listed is relevant to your study. If you are not sure if a list item applies to your research, read the appropriate section before selecting a response.

Materials & experimental systems

n/a	Involved in the study
	[x] Antibodies
	[x] Eukaryotic cell lines
[x]	Palaeontology and archaeology
	[x] Animals and other organisms
[x]	Clinical data
[x]	Dual use research of concern
[x]	Plants

Methods

n/a	Involved in the study
[x]	ChIP-seq
[x]	Flow cytometry
[x]	MRI-based neuroimaging

Antibodies

Antibodies used

Mouse-anti-E-Cadherin (1:200, BD Biosciences, # 610181)
Mouse-anti-E-Cadherin-488 (1:200, BD Biosciences, # 560061)
Rabbit-anti-Cytokeratin 5-Alexa488 (1:300, Abcam, # ab193894)
Mouse-anti-p63 (4A4) (1:200, Abcam, # ab735)
Rabbit-anti-Cytokeratin 7 [EPR17078] (1:8000, Abcam,#ab181598)
Rabbit-anti-Cytokeratin 7-Alexa Fluor 555[EPR17078] (1:300, Abcam, #ab209601)
Rabbit-anti-Cytokeratin 8 (1:200, Abcam, # ab59400)
Mouse-anti-Mucin 5AC antibody [45M1] (1:500, Abcam, #ab212636)
Rabbit anti-Cytokeratin 17[EP1623] (1:200, Abcam, #ab109725)
Mouse-anti-c-Jun [3/Jun] (1:1000, Abcam, #ab280089)
Mouse anti-Cytokeratin 6 [Ks6.KA12] (1:50, Abcam, #ab18586)Rabbit
Goat anti-gata6 (1:50, R&D systems, #AF1700-SP)
Rabbit anti-sox2(1:100, Abcam, #ab92494)
Rabbit anti-postn (1:1000, Invitrogen, #IPA5-79850)
Mouse anti-acta2(1:500, Abcam, #ab7817)
Rat anti-CD140a (PDGFRA)(APA5), (1:50, Invitrogen, #14-1401-82)
Rabbit anti-lor (1:50, Abcam, #ab85679)
Rabbit anti-CHGA (1:200, Abcam, #ab45179)
Donkey-anti-rabbit-Cy3 (1:150, Jackson ImmunoResearch, # 711-166-152)
Donkey-anti-rabbit-Alexa-647 (1:150, Jackson ImmunoResearch, # 711-605-152)
Donkey-anti-mouse -Cy5(1:150, Jackson ImmunoResearch, # 715-175-151)
Donkey-anti-goat-Cy3 (1:150, Jackson ImmunoResearch, # 705-165-147)
Donkey-anti-rat -Cy5(1:50, Jackson ImmunoResearch, #712-175-150)

Validation

All antibodies are commercially available and validation experiments for the respective antibodies were performed by the commercial manufacturer and below we provide the respective link for each antibody:
Mouse-anti-E-Cadherin
https://www.bdbiosciences.com/en-de/products/reagents/microscopy-imaging-reagents/immunofluorescence-reagents/purified-mouse-anti-e-cadherin.610181
Mouse-anti-E-Cadherin-488
https://www.bdbiosciences.com/en-de/products/reagents/microscopy-imaging-reagents/immunofluorescence-reagents/alexa-fluor-488-mouse-anti-e-cadherin.560061
Rabbit-anti-Cytokeratin 5-Alexa488
https://www.abcam.com/alexa-fluor-488-cytokeratin-5-antibody-ep1601y-ab193894.html
Mouse-anti-p63
https://www.abcam.com/p63-antibody-4a4-ab735.html
Rabbit-anti-Cytokeratin 7
https://www.abcam.com/products/primary-antibodies/cytokeratin-7-antibody-epr17078-cytoskeleton-marker-ab181598.html
Rabbit-anti-Cytokeratin 7-Alexa Fluor 555
https://www.abcam.com/products/primary-antibodies/alexa-fluor-555-cytokeratin-7-antibody-epr17078-ab209601.html
Rabbit-anti-Cytokeratin 8
https://www.abcam.com/cytokeratin-8-antibody-ab59400.html
Mouse-anti-Mucin 5AC
https://www.abcam.com/products/primary-antibodies/mucin-5ac-antibody-45m1-bsa-and-azide-free-ab212636.html
Rabbit anti-Cytokeratin 17
https://www.abcam.com/products/primary-antibodies/cytokeratin-17-antibody-ep1623-cytoskeleton-marker-ab109725.html
Mouse-anti-c-Jun
https://www.abcam.com/products/primary-antibodies/c-jun-antibody-3jun-ab280089.html
Mouse anti-Cytokeratin 6
https://www.abcam.com/products/primary-antibodies/cytokeratin-6-antibody-ks6ka12-ab18586.html
Goat anti-gata6
https://www.rndsystems.com/products/human-gata-6-antibody_af1700
Rabbit anti-sox2

https://www.abcam.com/en-dk/products/primary-antibodies/sox2-antibody-epr3131-ab92494
Rabbit anti-postn
https://www.thermofisher.com/antibody/product/Periostin-Antibody-Polyclonal/PA5-79850
Mouse anti-acta2
https://www.abcam.com/en-dk/products/primary-antibodies/alpha-smooth-muscle-actin-antibody-1a4-ab7817#all
Rat anti-CD140a (PDGFRA)(APA5)
https://www.thermofisher.com/antibody/product/CD140a-PDGFRA-Antibody-clone-APA5-Monoclonal/14-1401-82
Rabbit anti-lor
https://www.abcam.com/en-dk/products/primary-antibodies/loricrin-antibody-ab85679
Rabbit anti-CHGA
https://www.abcam.com/en-dk/products/primary-antibodies/chromogranin-a-antibody-ab45179
Donkey-anti-rabbit-Cy3
https://www.jacksonimmuno.com/catalog/products/711-166-152
Donkey-anti-rabbit-Alexa-647
https://www.jacksonimmuno.com/catalog/products/711-605-152
Donkey-anti-Mouse -Cy5
https://www.jacksonimmuno.com/catalog/products/715-175-151
Donkey-anti-goat-Cy3
https://www.jacksonimmuno.com/catalog/products/705-165-147
Donkey-anti-rat -Cy5
https://www.jacksonimmuno.com/catalog/products/712-175-150

Eukaryotic cell lines

Policy information about cell lines and Sex and Gender in Research

Cell line source(s)	3T3-J2 cells (kind gift from Craig Meyers; Howard Green laboratory, Harvard University)
Authentication	RRID: CVCL_W667
Mycoplasma contamination	Cell line was tested negative for the Mycoplasma contamination
Commonly misidentified lines (See ICLAC register)	NA

Animals and other research organisms

Policy information about studies involving animals; ARRIVE guidelines recommended for reporting animal research, and Sex and Gender in Research

Laboratory animals	For the study, 4 to 20 weeks female mice were used. The following genetic strains were used.<br>Wild type C57BL/6<br>Krt5-CreERT2; Rosa26-tdTomato<br>Krt8-CreERT2; Rosa26-tdTomato<br>Axin2-CreERT2;Rosa26-tdTomato<br>The animals were housed in autoclaved micro-isolator cages, where they had access to sterile drinking water and chow ad libitum. Mice were bred within the animal care facility, maintaining a 12-hour light/12-hour dark cycle, and ensuring a controlled environment with a temperature of 22.5±2.5 °C and humidity at 50±5%.
Wild animals	Study did not involve wild animals
Reporting on sex	Only female mice were used in this study.
Field-collected samples	No field collected samples were used.
Ethics oversight	All animal procedures were approved by the national legal and institutional authorities (Landesamt fur Gesundheit and Soziales (LaGaSo), Berlin, Germany, G 0026/17) at Max Planck Institute for Infection Biology, Berlin, Germany.

Note that full information on the approval of the study protocol must also be provided in the manuscript.
